# The impact of the distribution method for struvite (Crystal Green) on the chemical composition of soybean and their utility in animal nutrition

**DOI:** 10.1038/s41598-024-51625-3

**Published:** 2024-01-11

**Authors:** Anna Szuba-Trznadel, Anna Jama-Rodzeńska, Bernard Gałka, Rafał Ramut, Zygmunt Król, Daniel Jarki, Dragana Latković

**Affiliations:** 1 Department of Animal Nutrition and Feed Science, Faculty of Biology and Animal Science, Wroclaw University of Environmental and Life Sciences, 51-630 Wroclaw, Poland; 2 Institute of Agroecology and Plant Production, Faculty of Life Sciences and Technology, Wroclaw University of Environmental and Life Sciences, 50-363 Wroclaw, Poland; 3 Institute of Soil Science, Plant Nutrition and Environmental Protection, Faculty of Life Sciences and Technology, Wroclaw University of Environmental and Life Sciences, 50-363 Wroclaw, Poland; 4 Saatbau Poland Sp. z o.o., Żytnia 1, 55-300 Środa Śląska, Poland; 5 Department of Field and Vegetable Crops, University of Novi Sad, 21000 Novi Sad, Serbia

**Keywords:** Environmental impact, Agroecology

## Abstract

One of the main factors considered in assessing the nutritional value of feed is its chemical composition, which can be modified by fertilization. Faced with reducing P resources, alternative sources of this element are being sought. Phosphorus is an essential nutrient in soybean cultivation. The aim of the study was to use an alternative source of phosphorus fertilizer and compare its impact on the chemical composition of soybean seeds with that of a traditional fertilizer (Super FOS DAR). The study investigated a range of factors in animal nutrition as well as the basic content of macro- and microelements. A pot experiment with the Abelina soybean variety was conducted at the Experimental Station of the Wroclaw University of Environmental and Life Sciences. The experiment considered two factors against the control: phosphorus fertilizer placement (band, broadcast) and different phosphorus fertilization (Super FOS DAR, Crystal Green). Use of struvite (Crystal Green)) caused positive changes in selected amino acids content and in the nutritional value of protein in soybean seeds; this can enhance the value of soybean seeds as well as increase certain macroelements and microelements. Phosphorus fertilizer significantly increased the content of lysine, leucine, valine, phenyloalanine and tyrosine. Band fertilization with struvite caused a significant increase in amino acids (lysine, leucine, valine, phenyloalanine and tyrosine) as well as in the nutritional value of protein (as measured by the essential amino acid index, protein efficiency ratio and biological value of the protein). Favorable changes under the influence of the application of struvite were recorded in the content of calcium, as well as phosphorus, iron, and manganese. The value of the struvite in the case of its use as phosphorus fertilizer is promising; however, it needs further study.

## Introduction

Globally, soybean (*Glycine max* (L.) Merr.) is one of the most important legume crops as well as being an oilseed^1^. Currently, soybean oil is the second most popular oil in the world^2,3^, and the by-product of its production, i.e. extracted soybean meal, is the main source of protein in monogastric animal nutrition in Europe^4^.

The European Union (EU) is the largest importer of soybean meal. Soybean meal accounts for 70% of the high-protein components used in the production of compound feeds. Therefore, large imports of soybean meal to Europe have resulted in dependence on protein imports. So, with the aim of becoming independent of such imports, the EU is supporting development of domestic protein cultivation, including regional soybean crops. In this regard, attempts to introduce soybean cultivation in Polish conditions seems promising. The success of this crop cultivation in Poland would have the beneficial consequence of affording at least partial independence from the purchase of raw material from outside the EU^5,6^. Many factors determine the success of soybean cultivation, including the variety, weather conditions or any agrotechnical treatments required during the growing season of the plant, especially fertilization. The response of soybeans to nutrients depends on the source of fertilizer. When fertilizers are not properly applied, nutrients can be lost due to environmental conditions and poor management practices^7^.

Phosphorus (P) is the main essential nutrient for better growth, yield and various metabolic processes. It is involved in a wide range of plant processes, from enabling cell division to the development of a good root system. It stimulates pod setting, seed formation and protein synthesis. Enhancement of symbiotic nitrogen (N) fixation by root nodules as a result of P nutrition is supported by the high P requirements of the bacteria^8–10^. Either, the most important, in nutritional terms, is the protein content. Soybean is characterized by good quality protein (it contains about 380–450 g kg^−1^) with a high degree of digestibility and a rich composition of essential amino acids (EAA), mainly lysine (LYS)(which is particularly important in the feeding of monogastric animals)^11^. Hence, soybeans used in compound feeds complement the biological value of the protein found in cereal crops^12^. The amino acid composition of soybean protein is similar to that of animal protein, especially the content of EAA^13–15^.

Soybean seeds contain about 180–230 g kg^−1^ of oil. Globally, soybean is primarily considered a raw material for oil production^16,17^. It is generally accepted that the average fat content is 20%^18^. Soybean is characterized by a favourable composition of fatty acids, of which linoleic acid predominates^19,20^. A high content of essential fatty acids (the average level of EFAs is about 50% of the total acid pool) is necessary for the proper growth and development of animals^17,21^. Soybean seeds are a source of many valuable minerals (P, K, Ca, Mn, Zn, Fe and B)^11,20^.

Most agricultural fields are P-poor, and the consequent need to increase P content is leading to a rapid depletion of P supplies; this is a serious global problem^22,23^. A disturbance in the balance of elements in the soil can change the dynamics of other nutrients in plant parts by affecting their uptake and assimilation^24^. A faster release of P into the soil increases the uptake of this nutrient by plants^25,26^. However, excessive P fertilization can decrease the content of protein in seeds^27^. Improving the efficiency of P use in soybean cultivation in the form of fertilizers produced from sewage sludge could potentially contribute to reducing the use of chemical fertilizers and not adversely affect the agricultural environment^28^.

Struvite is considered a more environmentally friendly fertilizer compared to traditional fertilizers due to its slow release of nutrients into the soil, which reduces P leaching, contributing to increased yields even one year after its application^29^. Not only phoshorus fertilization but also the manner of its placement can affect yield, chemical composition and economic efficiency. Choosing the most appropriate method of applying P is complicated because of the many factors that affect P. Knowing the level of P via a soil test is important when determining placement options. An optimal approach located between band and broadcast fertilization is difficult to achieve^30,31^.

As a null hypothesis, we assume that there is no cause-and-effect relationship between the struvite and the nutritional value of soybean seeds in cases where the struvite is a substitute for superphosphate.

As a working hypothesis, we assume that STR fertilization will have a comparable or even higher effect on modifying the chemical composition of soybean seeds than superphosphate (SUP) because STR additionally contains N and Mg.

The aim of the study was to determine the effect of STR (Crystal Green) as a source of P produced from sewage sludge on the chemical composition of the Abellina seeds and its suitability in animal feeding.

## Materials and methods

### Experiment design

In 2022, a pot experiment was established at the Experimental Station of Wroclaw University of Environmental and Life Sciences (Pawlowice; geographical location 17°7′ E and 51°08′ N in the Lower Silesian Voivodship, Wrocław, Poland); this experiment incorporated the application of fertilizer produced from sewage sludge (Crystal Green) in soybean cultivation^32^. The experiment comprised two factors. Each variant with the factor was replicated six times. Varying placement of P fertilizer (band and broadcast) was the first factor of the experiment. Broadcast fertilization relied on random placement of fertilizers on the surface of the pot, while band fertilization involved placement of fertilizer granules at a depth of about 5 cm below the sown soybean seed. Traditional triple superphosphate (SUP) commonly used in soybean cultivation and STR (Crystal Green) was used in the experiment. The composition of Super FOS DAR fertiliser was 40% P_2_O_5_ (phosphorus pentoxide soluble in mineral acids), 25% P_2_O_5_ (soluble in neutral citrate solution and water), and 10% CaO (calcium oxide soluble in water).

Struvite contains N (2%), P (24%), and Mg (10%) and has a low heavy metal content compared to triple superphosphate^33^. From the chemical point of view, it is not pure struvite. STR was used in the form of granules with a diameter of about 1–2 mm. Fertilizer doses in the experiment were based on the optimum for growing soybeans under field conditions, i.e., a starting nitrogen dose of 30 kg ha^−1^ N, 70 kg ha^−1^ P_2_O_5_, and 120 kg ha^−1^ K_2_O. Nitrogen (N) and potassium (K) were used at this dose. The volume of the pot was around 5000 cm^3^. The following fertilizer doses per pot (converted) were applied:

The particle size distribution of the mineral parts corresponded to sandy clay. The soil was characterized by the Egner–Rhiem method as follows: P—10.3, K—22, and Mg—3.8 mg∙kg^−1^ d.m. Thus, K content was high, P average, and Mg low according to the soil abundance scale in force in Poland^34^ (Table 1).

**Table 1:** Doses of fertilizers used in the experiment.

The following fertilizer doses per pot (converted) were applied (in g)			
Struvite	Superphosphate	Ammonium nitrate	Potassium salt
0.76	0.54	0.27	1.25

In the 6th May 2022, Abelina variety seeds were sown into pots of six. The soybean seeds were sourced from Saatbau Polska Sp. z o.o.^35^. The seeds have been factory inoculated in Fix fertig technology.

This variety belongs to the “000” earliness group and is characterized by resilient early vigor. It is characterized by high yield potential with high stability in subsequent crop years. According to Research Centre for Cultivar Testing COBORU, it is also characterized by high fat (24.4%) and protein (36.9%) content. Prior to sowing, germination capacity was evaluated based on current standards^34^. The germination capacity of the tested variety averaged 75%. The number of seeds sown per pot was based on the optimal density of soybean seeds under the conditions.

During the growing season, observations were made for the presence of pests, diseases, and weeds as well as for the determination of developmental stages. Soybeans were watered regularly.

### Chemical analysis

Samples for chemical analysis were taken after harvesting. The concentration of nutrients was determined in a laboratory at the Department of Animal Nutrition and Feed Science, Wroclaw University of Environmental and Life Sciences, Poland.

- The dry matter** (**DM, AOAC: 934.01**)** for laboratory samples was examined by the gravimetric method at 105 °C for 4 h according to Polish standards. The chemical composition of the green fodder was assessed according to the Official Methods of Analysis of AOAC International (AOAC)^36^.
- Crude protein (CP, Kjeldahl method, AOAC: 984.13)—crude protein content was assessed by multiplying the nitrogen percentage (N %) determined in the sample using a Kjeltec 2300 Foss Tecator apparatus (AOAC: 984.13) with a factor (6.25). In order to reliably check the coverage of animals’ protein requirements, it is essential to determine the “true protein” (TP), defined as protein nitrogen (PN) obtained after separation of the non-protein nitrogen fraction (NPN) from total nitrogen (TN): TP = CP − NPN. True protein was separated from non-protein compounds by precipitation with 10% trichloroacetic acid and determined by the Bernstein method (AOAC: 920.154)^37^.
- Crude ash (CA, AOAC: 942.05)—crude ash was investigated by combustion of the sample in a muffle furnace (Czylok Company, Jastrzębie-Zdrój, Poland) at 550 °C for 24 h.
- Ether extract (EE, Soxhlet method comprising the extraction with ethyl ether, AOAC: 920.39A) was analyzed with the use of a BUCHI Extraction System B-811 (BÜCHI, Flawil, Switzerland).
- Crude fiber (CF) was analyzed by the Hennenberg–Stohman method (AOAC: 978.10) using a Fibertec Tecator Foss apparatus for laboratory analysis. Mineralization of the samples was determined with a Mars 5 version 194A06 (CEM Corporation, Matthews, NC, USA) microwave mineralization system using HNO3. Neutral detergent fiber (NDF) and acid detergent fiber (ADF) levels were determined according to the Van Soest methods (1991) using an Ankom 200 Fiber Analyzer (Ankom Technology Corporation, NY, USA).
- Gross energy (GE) of the seed was determined in a calorimetric bomb-calorimeter KL-11 “Mikado” (Precyzja-Bit Sp. z o.o., Bydgoszcz, Poland).
- Nitrogen-free extracts (NFE) content was calculated on the basis of difference according to the formula:
  $${\rm NFE} = {\rm dry\, matter} - ({\rm total\, protein} + {\rm crude \, fiber} + {\rm crude \, fat} + {\rm crude \, ash})$$
- Amino acids (AA) were determined by estimating by ion-exchange chromatography using an Amino Acids Analyzer AAA 400 (INGOS, Prague, the Czech Republic) according to standard AOAC protocols (AOAC: 994.12). Tryptophan was determined using a 2000 RS spectrophotometer (Aqualytic, Dortmund, German) at a wavelength of 590.0 nm (AOAC: 988.15).
- Macro-and microelements:
  - Nitrogen by the Kjeldahl method, P by the vanadomolibdate method, magnesium with titanium yellow, and potassium and calcium on a flame photometer (BWB Technologies UK Ltd., Newbury, UK) using flame photometry. Mineralization of plant material was completed using sulfuric acid and perhydrol in an electric furnace at 400 °C.
  - The microelements by atomic absorption spectrophotometry in a flame on a Spectra AA 200 apparatus by Varian. To determine each of the examined microelements, the required conditions concerning wavelength, slot width, and flame height were used.

Evaluation of the nutritional value of proteins:

The chemical score for restrictive amino acids (CS), the essential amino acid index (EAAI), and the protein efficiency ratio (PER) were used to assess the nutritional value of the protein.

The chemical score for restrictive amino acids was calculated based on the procedure given by Block and Mitchell (1945)^38^, which involves determining the ratio of the exogenous limiting amino acid content of the protein under study (a_b_) to the content of the same amino acid in the standard protein (a_a_):

$${\text{CS }} = {\text{ a}}_{{\text{b}}} /{\text{a}}_{{\text{a}}} \times { 1}00$$

In the research we use the whole egg protein standards (WE) as an amino acids standard^39^.

The essential amino acids index (EAAI) according to Oser (1951)^40^ was calculated as the geometric average of all EAA and histidine in relation to the content of these amino acids in the standard protein (in g per 100 g of CP). The index was calculated based on the formula:

$${\text{EAAI }} = { 1}0^{{{\text{logEAAI}}}}$$

The predicted protein efficiency ratio (PER) was calculated using the regression equations given by Alsmeyer et al. (1974)^41^:

$$\begin{gathered} {\text{PER1 }} = \, - 0.{684 } + \, 0.{456 } \times {\text{ Leu }} - \, 0.0{47 } \times {\text{ Pro}} \hfill \\ {\text{PER2 }} = \, - \, 0.{468 } + \, 0.{454 } \times {\text{ Leu }} - \, 0.{1}0{5 } \times {\text{ Tyr}} \hfill \\ {\text{PER3 }} = \, - { 1}.{816 } + \, 0.{435 } \times {\text{ Met }} + \, 0.{78}0 \, \times {\text{ Leu }} + \, 0.{211 } \times {\text{ His }} - \, 0.{944 } \times {\text{ Tyr}} \hfill \\ \end{gathered}$$

The biological value of the protein was calculated according to the equation given by Oser (1959)^42^:

$${\text{BV }} = { 1}.0{9 }\left( {{\text{EAA}}} \right) \, - { 11}.{7}$$

### Statistical analysis

Data from chemical analyses were subjected to Anova/Manova statistical analysis in Statistica software (version 13.1, StatSoft, Poland). The level of significance was α = 0.05. One-way and two-way analyses of average were performed to determine the effects of P fertilizer on the chemical analysis of soybean seeds. A principal component analysis (PCA) was applied to classify and segregate the different samples. Correlations were prepared using Statistica software (version 13.1, StatSoft, Poland)^43^. Standard deviations were added. Homogenous groups were calculated using a post hoc test.

### Ethics

Experimental research and field studies on plants (either cultivated or wild), including the collection of plant material, are fully compliant with the relevant institutional, national, and international guidelines and legislation. I declare that the plant material used for our study was purchased from Saatbau Polska Sp. z o.o. in Środa Śląska (Poland) as seed material. We did not use endangered plant species for the experiments.

## Results

### Nutritional value of soybean under phosphorus fertilization

The method of fertilizer application affected the chemical composition of soybean seeds. The phosphate fertilizer placement methods used had a significant effect only on CF content and non-protein nitrogen compounds (NPM) (Table 2). Significantly more CF was found when fertilizers were placed brodcast, while for NPM this was the case with band fertilization.

**Table 2:** The effect of phosphorus fertilization on the yield of protein and fat and on nutritional value in soybean seeds.

Method of fertilizer placement	Phosphorus fertilizer	DM	CA	CP	TP	NPN	CF	EE	NFE	GE	Yield	
											Protein	Fat
		%								MJ	kg ha^−1^	
Band		91.56 ± 0.46	6.52 ± 0.19	32.17 ± 0.44	30.99 ± 0.45	1.19a ± 0.10	5.74a ± 0.30	21.12 ± 0.89	26.01b ± 0.97	19.74 ± 0.10	1321 ± 203.30	897 ± 144.56
Broadcast		91.71 ± 0.46	6.56 ± 0.19	33.07 ± 0.44	31.961 ± 0.45	1.09b ± 0.10	6.41b ± 0.30	22.12 ± 0.89	23.55a ± 0.97	19.74 ± 0.10	1359 ± 203.30	940 ± 144.56
*P* value		n.s	n.s	n.s	n.s	**0.05**	**0.01**	n.s	**0.000**	ns	ns	ns
	Control	91.41 ± 0.51	6.39a ± 0.03	33.27 ± 1.85	32.10 ± 1.89	1.17 ± 0.12	5.75 ± 0.25	21.68 ± 0.44	24.34 ± 2.09	19.80b ± 0.07	1377 ± 183.3	923 ± 91.36
	SUP	91.60 ± 0.51	6.51a ± 0.03	32.68 ± 1.85	31.55 ± 1.89	1.14 ± 0.12	6.32 ± 0.25	22.06 ± 0.44	24.08 ± 2.09	19.74ab ± 0.07	1297 ± 183.3	911 ± 91.36
	STR	91.80 ± 0.51	6.73b ± 0.03	31.91 ± 1.85	30.77 ± 1.89	1.09 ± 0.12	6.19 ± 0.25	22.11 ± 0.66	25.92 ± 2.09	19.68a ± 0.07	1347 ± 183.3	921 ± 91.36
*P* value		n.s	**0.01**	n.s	n.s	n.s	n.s	n.s	ns	**0.05**	ns	ns
Band	Control	91.54 ± 0.33	6.47ab ± 0.03	33.87c ± 0.03	32.67c ± 0.15	1.21 ± 0.18	5.05a ± 0.39	21.08a ± 0.66	25.14 ± 1.38	19.75abc ± 0.09	1632c ± 12.41	1055b ± 25.79
Band	SUP	91.29 ± 0.33	6.36a ± 0.03	32.38b ± 0.03	31.22b ± 0.15	1.17 ± 0.18	6.05b ± 0.39	21.41ab ± 0.66	25.28 ± 1.38	19.81bc ± 0.09	1094a ± 12.41	740a ± 25.79
Band	STR	91.75 ± 0.33	6.74c ± 0.03	30.25a ± 0.03	28.95a ± 0.15	1.16 ± 0.18	6.16b ± 0.39	23.35c ± 0.66	27.62 ± 1.38	19.67a ± 0.09	1237ab ± 12.41	896ab ± 25.79
Broadcast	Control	91.29 ± 0.33	6.31a ± 0.03	32.68 ± 0.03	31.65b ± 0.15	1.13 ± 0.18	6.45b ± 0.39	22.48bc ± 0.66	23.55 ± 1.38	19.86c ± 0.09	1122a ± 12.41	791a ± 25.79
Broadcast	SUP	91.91 ± 0.33	6.66bc ± 0.03	32.98ab ± 0.03	31.89bc ± 0.15	1.10 ± 0.18	6.64b ± 0.39	23.00c ± 0.66	22.89 ± 1.38	19.67a ± 0.09	1500bc ± 12.41	1082b ± 25.79
Broadcast	STR	91.85 ± 0.33	6.72bc ± 0.03	33.55ab ± 0.03	32.50c ± 0.15	1.03 ± 0.05	6.12b ± 0.39	20.96a ± 0.66	24.22 ± 1.38	19.71ab ± 0.09	1456abc ± 12.41	947ab ± 25.79
*P* value		n.s	**0.01**	**0.001**	**0.001**	n.s	**0.01**	**0.001**	ns	**0.002**	**0.000**	**0.000**

Values with different letters differ significantly.

*DM* dry matter, *CA* crude ash, *CP* crude protein, *TP* true protein, *NPN *non-protein nitrogen fraction, *CF* crude fibre, *EE* ether extract, *NFE *nitrogen-free extracts, *GE* gross energy.

Significant values are in bold.

The results are consistent with the working hypothesis that fertilization with STR would significantly increase the CA content. The highest CA content was found after STR application (6.73%) regardless of the fertilizer application site (*p* < 0.01). Seed content of other nutrients did not varry according to the source of P fertilization. The interaction of factors caused a modification in the majority of parameters and affected the nutritional value of soybean seeds, such as CA, CP, TP, CF, and EE content, and also GE value (Table 2).

Struvite fertilization, both band and broadcast, contributed to an increase in CA content, while it did not significantly increase CP content. The situation is different for TP. Broadcast fertilization with STR caused an increase in TP content.

According to our working hypothesis, phosphorus fertilization significantly affected protein value. Indeed, the lowest CP and TP values were recorded when STR was applied band. On the other hand, when STR was applied broadcast, CP and TP values were the highest (*p* < 0.001). When the fertilizer was applied broadcast, the proportion of TP was 97% and N-PNF was 3% in CP; however, when the fertilizer was applied band, the proportion of TP was 96% and N-PNF was 4% in CP.

**Figure 1:**
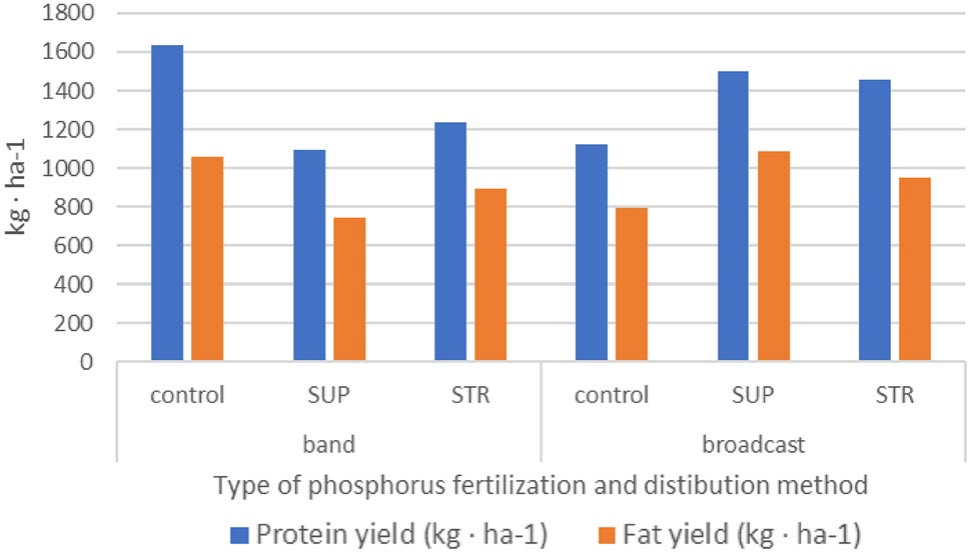
The effect of phosphorus fertilization on the yield of protein and fat in the seeds of soybean. Control—without phosphorus fertilization; SUP—triple superphosphate as Super FOS DAR 40; *STR—*struvite as Crystal Green; band—P fertilizer placed 5 cm below seeds; broadcast—P fertilizer placed on surface of the soil.

In addition to CP values, fat content (EE) is an also an important characteristic. Soybean seeds are characterized by high CP (over 30%) and EE (over 20%). Analyzing the fat content (EE) of the seeds studied, a different correlation was found than that for protein (CP). Thus, EE was higher where STR was placed band, and lower where it was applied broadcast. Fat content affects gross energy in the seeds (the greater the gross energy, the higher the fat content). These results were reflected in the yield. Protein and fat yield were not dependent on the placement of phosphorus fertilizers or its type. Interaction between factors affected the protein yield with a positive tendency, while struvite application regardless of the its placement also affected this characteristic.. Analyzing the results, it can be assumed that broadcast application of P fertilization was insignificantly more advantageous compared to band application (Fig. 1).

The level of NFE compounds in the tested samples ranged from 23 to 28%. The content of this fraction was calculated on the basis of the determined basic chemical composition. This includes easily hydrolyzable sugars, such as simple sugars, disaccharides, polysaccharides (starch), and volatile fatty acids.

The biological value depends on the amount and type of fiber, among other factors. The crude fiber content ranged from 5.1% to 6.6%. Phosphorus fertilizer placement had a significant effect on this parameter. The highest content was found under broadcast fertilization.

There was a positive correlation between CF and EE (the higher the fat (EE) content, the higher the fiber (CF) content increases), and also between TC and CP (Table 3). A negative correlation was found for CP content and EE, as well as EE and TP.

**Table 3:** Relationships between chemical composition in soyabean seeds.

	Average	Standard error	EE	CP	TP	CF
EE	22.05	1.02	×			
CP	32.73	1.25	−0.57	×		
TP	31.47	1.30	−0.63	0.97	×	
CF	6.08	0.56	0.61	−0.26	−0.26	×

*EE* ether extract, *CP* crude protein, *TP* true protein, *CF* crude fibre.

**Figure 2:**
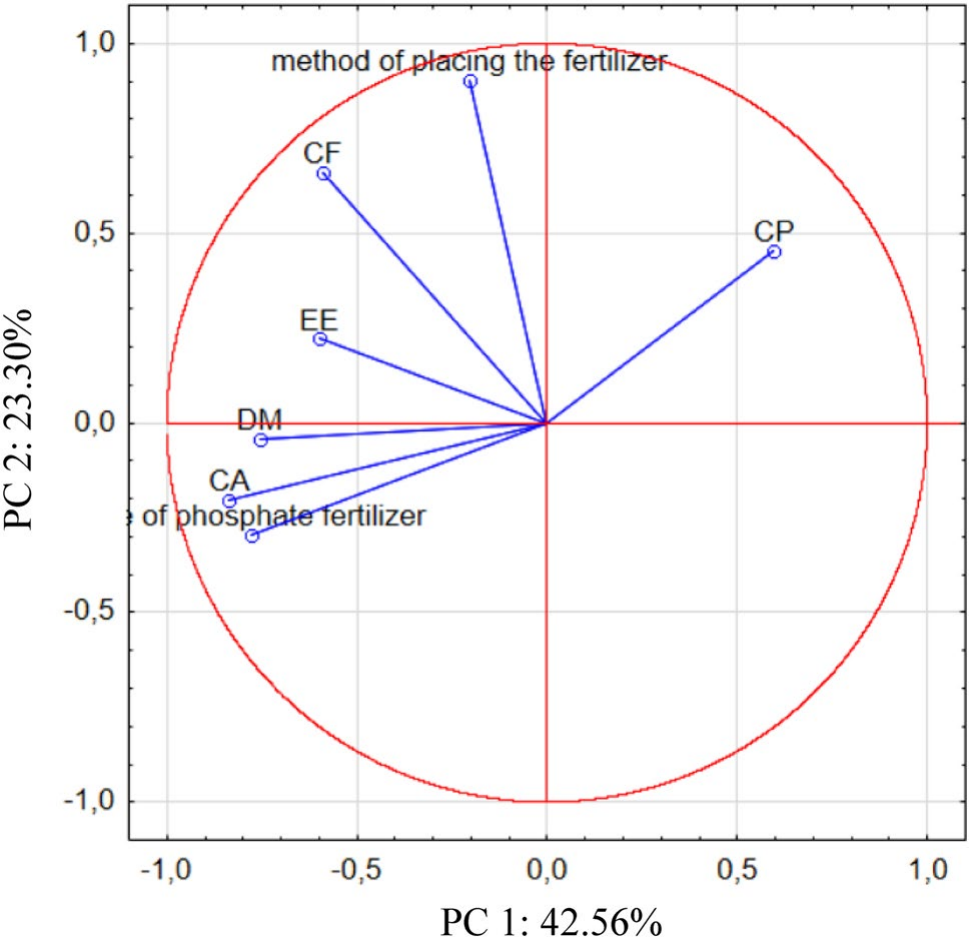
The PCA biplot diagram, showing the relationships among chemical composition of soybean seeds. Method of placing the fertilizer: band—P fertilizer placed 5 cm below seeds; broadcast—P fertilizer placed on surface of the soil. Type of fertilizers: SUP—triple superphosphate as Super FOS DAR 40; STR—struvite as Crystal Green. *DM* dry matter, *CP* crude protein, *CA* crude ash, *EE* ether extract, *CF* crude fibre.

To better explain the obtained chemical composition data for soybean seeds and similarities and differences observed between the samples, PCA was used, and the extracted results are presented in Fig. 2. The first PC explained 42.56%, and the second explained 23.30% of the total variance within the observed data.

### Amino acid composition of soybean under phosphorus fertilization

**Figure 3:**
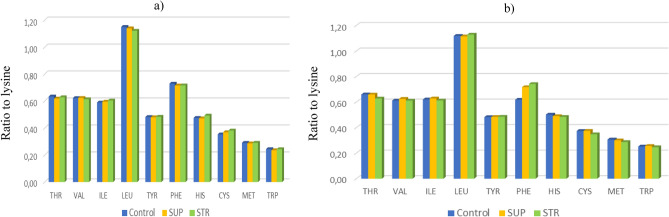
The essential amino acid profile relative to lysine: (**a**) band; (**b**) broadcast. Type of fertilizers: SUP—triple superphosphate as Super FOS DAR 40, STR—struvite as Crystal Green.

**Figure 4:**
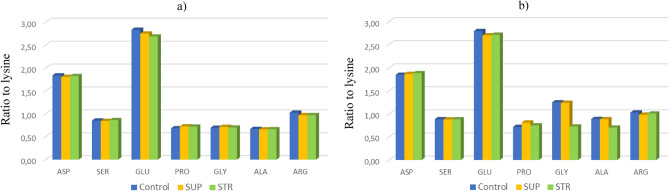
Non-essential amino acid profile relative to lysine: (**a**) band; (**b**) broadcast. Control—without phosphorus fertilization; SUP—triple superphosphate as Super FOS DAR 40; STR—struvite as Crystal Green.

Soybeans have a high protein content (ca 33% CP) and also a favorable amino acid profile (Tables 3 and 4). Taking into account the biological value of feed material, the content of amino acids is pivotal. This is especially the case for the content of EAA that are important in monogastric nutrition. This is why expression of the content of an amino acid relative to lysine is a conventional means of expressing dietary protein quality for monogastric animals (Figs. 3 and 4). Soybean seeds are a good source of LYS, although it is relatively poor in MET. The first amino acid limiting the biological value in our study was VAL. In order, the next limiting amino acid was ILE and the sulfur amino acids MET with CYS.

**Table 4:** The effect of phosphorus fertilization on essential amino acid content of soybean seeds (g per 100 g of CP).

Method of fertilizer placement	Phosphorus fertilizer	LYS	MET	CYS	THR	TRP	HIS	LEU	ILE	VAL	PHE	TYR
		g (100 g of CP)^−1^										
Band		5.95b ± 0.20	1.72 ± 0.07	2.19 ± 0.08	3.74 ± 0.12	1.44 ± 0.03	2.86 ± 0.07	6.77b ± 0.13	3.55 ± 0.08	3.69b ± 0.09	4.29b ± 0.31	2.87b ± 0.04
Broadcast		5.68a ± 0.20	1.69 ± 0.07	2.08 ± 0.08	3.69 ± 0.12	1.43 ± 0.03	2.79 ± 0.07	6.37a ± 0.13	3.53 ± 0.08	3.50a ± 0.09	3.93a ± 0.31	2.75a ± 0.04
*P* value		**0.01**	ns	ns	ns	ns	ns	**0.001**	ns	**0.01**	**0.01**	**0.05**
	Control	5.73 ± 0.29	1.71 ± 0.10	2.08 ± 0.22	3.71 ± 0.23	1.43 ± 0.06	2.80 ± 0.17	6.50 ± 0.32	3.47 ± 0.19	3.54 ± 0.19	3.86a ± 0.15	2.76 ± 0.14
	SUP	5.79 ± 0.29	1.70 ± 0.10	2.15 ± 0.22	3.70 ± 0.23	1.43 ± 0.06	2.78 ± 0.17	6.53 ± 0.32	3.55 ± 0.19	3.62 ± 0.19	4.15ab ± 0.15	2.79 ± 0.14
	STR	5.93 ± 0.29	1.71 ± 0.10	2.17 ± 0.22	3.73 ± 0.23	1.45 ± 0.06	2.90 ± 0.17	6.67 ± 0.32	3.61 ± 0.19	3.63 ± 0.19	4.32b ± 0.15	2.87 ± 0.14
*P* value		ns	ns	ns	ns	ns	ns	ns	ns	ns	**0.05**	ns
Band	Control	5.73a ± 0.02	1.66ab ± 0.02	2.02ab ± 0.02	3.64ab ± 0.10	1.40a ± 0.01	2.72a ± 0.003	6.59ab ± 0.11	3.38a ± 0.09	3.57ab ± 0.05	4.18b ± 0.04	2.76a ± 0.03
Band	SUP	5.92b ± 0.02	1.70abc ± 0.02	2.19c ± 0.02	3.67ab ± 0.10	1.40a ± 0.01	2.80b ± 0.003	6.76bc ± 0.11	3.53ab ± 0.09	3.69bc ± 0.05	4.24bc ± 0.04	2.84ab ± 0.03
Band	STR	6.19b ± 0.02	1.80c ± 0.02	2.37d ± 0.02	3.89b ± 0.10	1.51c ± 0.01	3.05d ± 0.003	6.95c ± 0.11	3.75b ± 0.09	3.80bc ± 0.05	4.44c ± 0.04	3.00b ± 0.03
Broadcast	Control	5.73a ± 0.02	1.75bc ± 0.02	2.14bc ± 0.02	3.78ab ± 0.10	1.45ab ± 0.01	2.87c ± 0.093	6.41a ± 0.11	3.56ab ± 0.09	3.50ab ± 0.05	3.54a ± 0.04	2.77a ± 0.03
Broadcast	SUP	5.65a ± a0.02	1.70abc ± 0.02	2.12bc ± 0.02	3.73ab ± 0.10	1.45b ± 0.01	2.77ab ± 0.003	6.31a ± 0.11	3.56ab ± 0.09	3.53ab ± 0.05	4.06b ± 0.04	2.74a ± 0.03
Broadcast	STR	5.66a ± 0.02	1.62a ± 0.02	1.97a ± 0.02	3.57a ± 0.10	1.40a ± 0.01	2.74ab ± 0.003	6.39a ± 011	3.47ab ± 0.09	3.46a ± 0.05	4.20b ± 0.04	2.75a ± 0.03
*P* value		**0.001**	**0.001**	**0.01**	**0.01**	**0.001**	**0.001**	**0.01**	**0.001**	**0.05**	**0.01**	**0.05**

Values with different letters differ significantly.

Significant values are in bold.

Fertilizer placement significantly affected LYS, LEU, VAL, PHE, and TYR. Significantly higher contents of these amino acids were found under band fertilization. Struvite significantly increased the content of PHE (Table 4). Band fertilization with STR caused a significant increase in ILE, LEU, TYR, HIS, LYS, CYS and PHY, MET.

The method of P fertilizer led to changes in GLU, GLY, ALA, and ARG content in the seeds (Fig. 4). A significantly higher content of GLY and ALA was found under broadcast fertilization, while GLU and ARG content increased with band fertilization. Phosphorus fertilizer had a significant impact only on ASP, PRO, and ARG content. Struvite caused an increase in ASP and ARG content, while it had no impact on PRO content. Interaction between the factors affected all the examined amino acids (Table 5). In the majority of cases, striuvite caused an increase in the amino acid content (ASP, SER, GLU, ARG) irrespective of fertilization method (Fig. 4).

**Table 5:** The effect of phosphorus fertilization on non-essential amino acid content of soybean seeds (g per 100 g of CP).

Method of fertilizer placement	Phosphorus fertilizer	ASP	SER	GLU	PRO	GLY	ALA	ARG
		g (100 g of CP)^−1^						
Band		10.84 ± 0.10	5.08 ± 0.10	16.40b ± 0.38	4.23 ± 0.08	4.19 ± 1.49	3.95a ± 0.53	5.88b ± 0.15
Broadcast		10.59 ± 0.10	5.00 ± 0.10	15.56a ± 0.38	4.31 ± 0.08	6.09 ± 1.49	4.69b ± 0.53	5.71a ± 0.15
*P* value		ns	ns	**0.001**	ns	**0.001**	**0.001**	**0.05**
	Control	10.55a ± 0.40	4.97 ± 0.23	16.40 ± 0.77	4.01a ± 0.19	5.57 ± 0.15	4.46 ± 0.16	5.87b ± 0.15
	SUP	10.63ab ± 0.40	4.98 ± 0.23	15.56 ± 0.77	4.45b ± 0.19	5.63 ± 0.15	4.46 ± 0.16	5.65a ± 0.15
	STR	10.98b ± 0.40	5.18 ± 0.23	16.02 ± 0.77	4.35b ± b0.19	4.23 ± 0.15	4.05 ± 0.16	5.86b ± 0.15
*P* value		**0.05**	ns	ns	**0.001**	ns	ns	**0.05**
Band	Control	10.53a ± 0.001	4.88a ± 0.05	16.24c ± 0.04	3.92a ± 0.04	3.98a ± 0.001	3.83a ± 0.04	5.87bc ± 0.02
Band	SUP	10.71a ± 0.001	5.01ab ± 0.05	16.31c ± 0.04	4.31bcd ± 0.04	4.21b ± 0.001	3.91a ± 0.04	5.75ab ± 0.02
Band	STR	11.30b ± 0.001	5.34b ± 0.05	16.65c ± 0.04	4.47 cd ± 0.04	4.35b ± 0.001	4.11a ± 0.04	6.02c ± 0.002
Broadcast	Control	10.58a ± 0.001	5.06ab ± 0.05	16.01bc ± 0.04	4.12ab ± 0.04	7.16c ± 0.001	5.08b ± 0.04	5.89bc ± 0.002
Broadcast	SUP	10.54a ± 0.001	4.97a ± 0.05	15.28a ± 0.04	4.58d ± 0.04	7.01c ± 0.001	4.99b ± 0.04	5.55a ± 0.002
Broadcast	STR	10.66a ± 0.001	4.99ab ± 0.05	15.38ab ± 0.04	4.25bc ± 0.04	4.11ab ± 0.001	3.98a ± 0.04	5.70ab ± 0.002
*P* value		**0.01**	**0.01**	**0.01**	**0.01**	**0.001**	**0.001**	**0.01**

Values with different letters differ significantly.

Significant values are in bold.

The proportion of GLU increased by 2.5% in the seeds where STR fertilization was applied band. However, when broadcast fertilizers were applied, there was a decrease in this amino acid: by about 5% (with SUP application) and by about 4% (with STR application), respectively. In line with our working hypothesis, fertilization also contributed to an increase in the proportion of PRO: by about 11% (with SUP application) and by about 8.5% (with STR application). The increase in less valuable protein fractions may have influenced the lower values of indices determining the biological value of soybean seed protein (Table 5).

Amino acids (mainly EAA) determine the biological value of a protein. The sum of all amino acids averaged 89.56 g per 16 g N. Phosphorus fertilizer application had a significant impact only on the sum of essential amino acids (Table 6), with a higher content under band placement. Phosphorus fertilization had no significant effect on any sum of amino acids. Interaction between factors significantly affected all determined parameters. Struvite application using the band method significantly increased the content of the sum of all amino acids, sum of essential amino acids and non-essential amino acids. On the other hand, phosphorus fertilizer application caused a decrease in the above paramaters.

**Table 6:** Nutritional value of protein and sum of amino acids in soybean seeds.

Method of fertilizer placement	Phosphorus fertilizer	CP	AA	EAA	non-EAA	CS	EAAI	∑ EAA	PER 1	PER 2	PER 3	BV
		g kg^−1^ DM	g per 100 g of CP									
Band		351.36 ± 4.60	89.67 ± 2.45	39.09b ± 0.28	50.59 ± 2.26	46.16 ± 1.04	71.8b ± 0.91	76.2b ± 0.83	2.20b ± 0.06	2.30b ± 0.06	2.10b ± 0.09	71.31b ± 0.99
Broadcast		360.62 ± 4.60	89.45 ± 2.45	37.48a ± 0.28	51.97 ± 2.26	45.85 ± 1.04	69.5a ± 0.91	73.8a ± 0.83	2.01a ± 0.06	2.13a ± 0.06	1.88a ± 0.09	68.70a ± 0.99
*P* value		ns	ns	**0.05**	ns	ns	**0.05**	**0.05**	**0.001**	**0.001**	**0.001**	**0.05**
	Control	364.06 ± 20.51	89.18 ± 4.05	37.60 ± 2.04	51.57 ± 2.04	45.05 ± 2.52	70.3 ± 3.37	73.9 ± 4.04	2.09 ± 0.14	2.19 ± 0.13	1.98 ± 0.19	68.88 ± 4.79
	SUP	356.38 ± 20.51	89.80 ± 4.05	38.21 ± 2.04	51.59 ± 2.04	46.06 ± 2.52	70.3 ± 3.37	74.6 ± 4.04	2.08 ± 0.14	2.20 ± 0.13	1.97 ± 0.19	69.67 ± 4.79
	STR	347.53 ± 20.51	89.71 ± 4.05	39.04 ± 2.04	50.67 ± 2.04	49.80 ± 2.52	71.6 ± 3.37	76.3 ± 4.04	2.16 ± 0.14	2.26 ± 0.13	2.03 ± 0.19	71.48 ± 4.79
*P* value		ns	ns	ns	ns	ns	Ns	ns	ns	ns	ns	Ns
Band	Control	369.87c ± 1.70	86.95ab ± 0.52	37.68ab ± 0.38	49.26a ± 0.14	43.9a ± 1.17	70.2a ± 0.76	73.4ab ± 0.54	2.13bc ± 0.05	2.23 ± 0.05	2.01abc ± 0.05	68.29ab ± 0.65
Band	SUP	354.81b ± 1.70	89.01abc ± 0.52	38.78b ± 0.38	50.24ab ± 0.14	45.9ab ± 1.17	70.8a ± 0.76	75.2b ± 0.54	2.19 cd ± 0.05	2.30 ± 0.05	2.10bc ± 0.05	70.32b ± 0.65
Band	STR	329.40a ± 1.70	93.06d ± 0.52	40.81c ± 0.38	52.26bc ± 0.14	48.7b ± 1.17	74.5b ± 0.76	79.9c ± 0.54	2.27d ± 0.05	2.37 ± 0.05	2.21c ± 0.05	75.33c ± 0.65
Broadcast	Control	358.26ab ± 1.70	91.41 cd ± 0.52	37.53ab ± 0.38	53.88c ± 0.14	46.2ab ± 1.17	70.3a ± 0.76	74.5ab ± 0.54	2.04ab ± 0.05	2.15 ± 0.05	1.94ab ± 0.05	69.46ab ± 0.65
Broadcast	SUP	357.95ab ± 1.70	90.58bcd ± 0.52	37.64 ± 0.38	52.94c ± 0.38	46.2ab ± 1.17	69.7a ± 0.76	74.1ab ± 0.54	1.98a ± 0.05	2.11 ± 0.05	1.84a ± 0.05	69.03ab ± 0.65
Broadcast	STR	365.67bc ± 1.70	86.36a ± 0.52	37.27a ± 0.380	49.08a ± 0.38	45.1ab ± 1.17	68.7a ± 0.76	72.8a ± 0.54	2.03ab ± 0.05	2.14 ± 0.05	1.87a ± 0.05	67.62a ± 0.65
*P* value		**0.001**	**0.01**	**0.001**	**0.001**	**0.01**	**0.001**	**0.001**	**0.05**	ns	**0.05**	**0.001**

Values with different letters differ significantly.

*CP* crude protein, *AA *sum of all amino acids, *EAA *sum of essential amino acids, *non-EAA *sum of non-essential amino acids, *CS* (chemicalscore)—the limiting amino acid index, *EAAI* the essential amino acids index; the predicted PER (protein efficiency ratio), *BV *the biological value of the protein.

Significant values are in bold.

The CS ratio averaged 46% according to WE. The essential amino acids (EAA) averaged 43% of the total of all amino acids, as reflected in the high essential amino acid index (EAAI). This index, determined according to the accepted standard, was the highest for the band application and amounted to 71.8%. Based on the calculated indices (expressing the biological value of the protein), it can be concluded that the application of band fertilization caused an increase in EAA compared to their content in the standard protein (WE).

The method of placing the fertilizer significantly affected the following parameters: EAAI, PER 1, PER 2, PER 3, and BV. Phosphorus fertilization had no significant effect on the determined parameters. Taking into account the interaction between factors, STR applied band caused an increase in all the evaluated parameters. The calculated predicted PERs (PER 1—2.27%; PER 2—2.37%; PER 3—2.21%) confirm the highest nutritional value for soy protein when STR was applied band. A positive correlation was found between the selected amino acids as shown in Table 7.

**Table 7:** Relationships between amino acid in soyabean seeds.

Variable	Correlations (amino acids) determined correlation coefficients are significant with p < 0.05000, N = 18 (missing data were removed by chance)													
	Average	Standard deviation (SD)	THR	VAL	ILE	LEU	TYR	PHE	PHE + TYR	HIS	LYS	CYS	MET	MET + CYS
THR	3.714773	0.139646	×											
VAL	3.598455	0.138099	0.550080	×										
ILE	3.542615	0.145667	0.902895	0.597535	×									
LEU	6.571586	0.253242	0.516734	0.885497	0.521990	×								
TYR	2.811304	0.110501	0.682005	0.600173	0.712639	0.766756	×							
PHE	4.112555	0.296866	0.061690	0.470334	0.212958	0.586565	0.590138	×						
PHE + TYR	6.923858	0.372904	0.251207	0.552275	0.380707	0.694169	0.766129	0.970964	×					
HIS	2.830020	0.117377	0.714170	0.596706	0.735483	0.549164	0.752038	0.168108	0.356677	×				
LYS	5.819333	0.204680	0.624343	0.910336	0.681251	0.924669	0.812659	0.512235	0.648598	0.777081	×			
CYS	2.137281	0.137617	0.775406	0.703615	0.751972	0.650188	0.815626	0.311596	0.489750	0.876191	0.815722	×		
MET	1.710779	0.070000	0.780790	0.349479	0.667954	0.388103	0.763145	0.044589	0.261636	0.821637	0.567361	0.866976	×	
MET + CYS	3.848061	0.201350	0.801411	0.602397	0.746167	0.579309	0.822766	0.228468	0.425688	0.884495	0.754767	0.984877	0.940206	×

**Figure 5:**
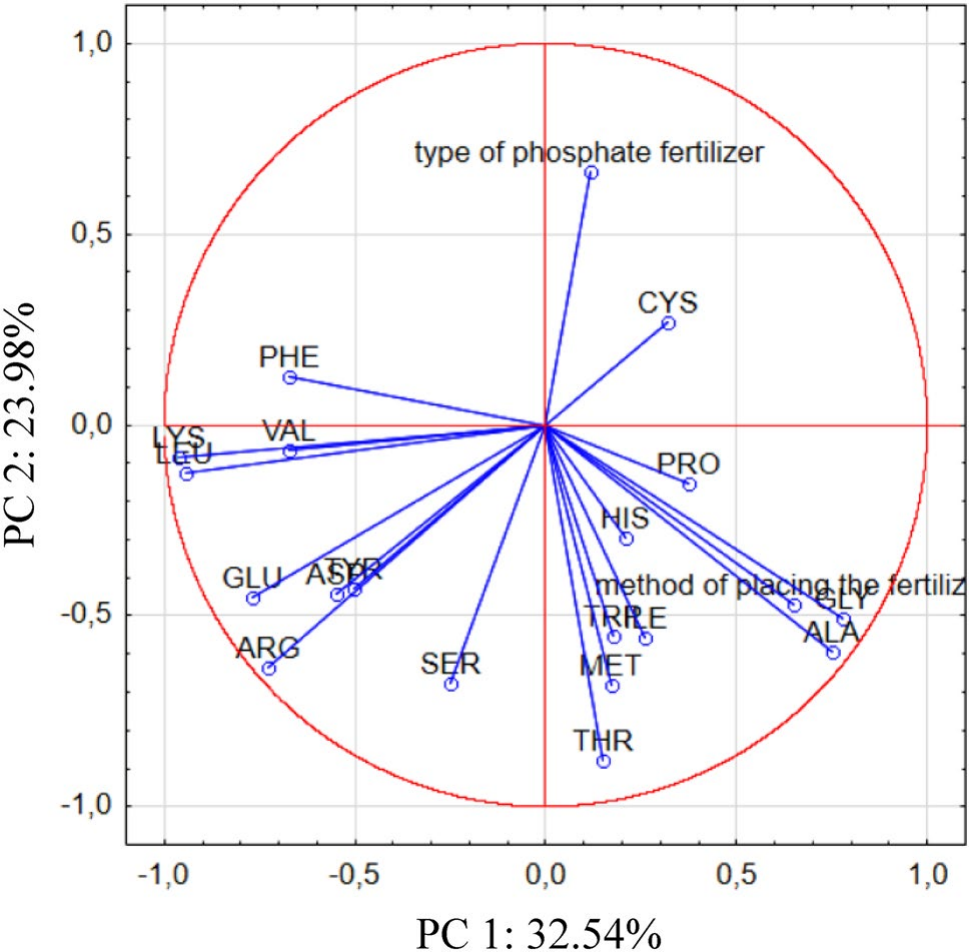
The PCA biplot diagram, showing the relationships among amino acid profiles of soybean seeds. Method of placing the fertilizer: band—P fertilizer placed 5 cm below seeds, broadcast—P fertilizer placed on surface of the soil. Type of fertilizers: SUP—triple superphosphate as Super FOS DAR 40, STR—struvite as Crystal Green. *ASP* aspartic acid, *THR* threonine, *SER* serine, *GLU* glutamic acid, *PRO* proline, *GLY* glycine, *ALA* alanine, *CYS* cystine, *VAL* valine, *MET* methionine, *ILE* isoleucine, *LEU* leucine, *TYR* tyrosine, *PHE* phenyloalanine, *HIS* histidine, *LYS* lysine, *ARG* arginine.

For the graphical representation of the amino acid profile data for soybean seeds across the samples, PCA was used, and the extracted results are presented in Fig. 5. The first PC explained 32.54%, and the second explained 23.98% of the total variance within the observed data.

### Macro- and microelements in soybean seeds under phosphorus fertilization

Placement of P fertilization had a significant effect on Ca, Na, and K content, with a higher content being noted with band fertilization. Phosphorus fertilization had a significant impact on Ca, Na, and P content in soybean seeds. In line with our working hypothesis, struvite caused an increase in P and Ca content. Interaction between factors had a significant impact on all examined elements. Broadcast fertilization with STR led to an increase in the N, Ca, and P content in soybean seeds (Table 8). Na content was found to be the highest in seeds when using in fertilization both while SUP and STR band fertilization.

**Table 8:** The effect of phosphorus fertilization on macroelements content (g·kg^−1^).

Method of fertilizer placement	Phosphorus fertilizer	N	Ca	Mg	Na	K	P
Band		51.47 ± 0.71	5.81a ± 1.46	2.72 ± 0.18	0.266b ± 0.18	17.09b ± 0.71	8.04 ± 0.48
Broadcast		52.91 ± 0.71	7.18b ± 1.46	2.69 ± 00.18	0.232a ± 0.18	14.36a ± 0.71	7.94 ± 0.48
P value		ns	**0.05**	ns	**0.01**	**0.001**	ns
	Control	53.24 ± 2.96	5.43a ± 1.08	2.73 ± 0.09	0.225 ± 0.13	16.21 ± 0.00	7.56a ± 0.08
	SUP	52.29 ± 2.96	6.82ab ± 1.08	2.61 ± 0.09	0.271 ± 0.12	15.31 ± 0.00	8.10b ± 0.08
	STR	51.05 ± 2.96	7.23b ± 1.08	2.74 ± 0.09	0.251 ± 0.12	15.68 6 ± 0.00	8.37 c ± 0.08
P value		ns	**0.01**	ns	**0.01**	ns	**0.001**
Band	Control	54.20a ± 0.06	5.62a ± 0.16	3.03e ± 0.01	0.229b ± 0.02	18.81e ± 1.03	7.76b ± 0.045
Band	SUP	51.81b ± 0.06	5.58a ± 0.16	2.53a ± 0.01	0.287d ± 0.02	16.89a ± 1.03	8.12b ± 0.045
Band	STR	48.41c ± 0.06	6.25b ± 0.16	2.93 d ± 0.01	0.282d ± 0.02	16.35d ± 1.03	8.31d ± 0.045
Broadcast	Control	52.29b ± 0.06	5.25a ± 0.16	2.43b ± 0.01	0.221a ± 0.02	14.59b ± 1.03	7.34a ± 0.045
Broadcast	SUP	52.78bd ± 0.06	8.09c ± 0.16	2.89c ± 0.01	0.256c ± 0.02	14.33c ± 1.03	8.08c ± 0.045
Broadcast	STR	53.69ad ± 0.06	8.22c ± 0.16	2.77c ± 0.01	0.220a ± 0.02	14.77c ± 1.03	8.44e ± 0.045
P value		**0.00**	**0.000**	**0.000**	**0.000**	**0.003**	**0.000**

Values with different letters differ significantly.

Significant values are in bold.

Placement of P fertilizer had a significant impact on Fe and Zn content. In line with our working hypothesis, phosphorus fertilization in the form of STR caused an increase in Fe and Mn content in soybean seeds. Interactions between factors, in the case of Cu, Fe, and Mn with strivite applied broadcast, increased the content of these elements. However, the highest content of Zn was found in those pots where SUP was applied band (Table 9).

**Table 9:** The effect of phosphorus fertilization on microelements content (mg·kg^−1^).

Method of fertilizer placement	Phosphorus fertilizer	Cu	Fe	Mn	Zn
Band		21.95 ± 1.23	100.32a ± 11.75	45.23 ± 6.35	72.52b ± 3.43
Broadcast		22.72 ± 1.23	111.07b ± 11.75	48.71 ± 6.35	67.01a ± 3.43
P value		ns	**0.05**	ns	**0.01**
	Control	22.28 ± 2.11	97.49 ± 14.57	45.50a ± 5.07	70.53 ± 2.34
	SUP	22.39 ± 2.11	108.13 ± 14.5	43.30a ± 5.07	69.73 ± 2.34
	STR	22.32 ± 2.11	112.96 ± 14.5	52.12b ± 5.07	69.04 ± 2.34
P value		ns	**0.05**	**0.00**	ns
Band	Control	22.23a ± 1.23	94.91a ± 4.58	43.98b ± 2.70	74.12d ± 2.21
Band	SUP	23.18c ± 1.23	109.25b ± 2.04	44.25b ± 2.21	76.13d ± 2.21
Band	STR	20.44a ± 1.23	99.80b ± 2.04	47.50c ± 2.21	67.32b ± 2.21
Broadcast	Control	22.34ab ± 1.23	100.06 ± 2.04	47.02c ± 2.21	66.95b ± 2.21
Broadcast	SUP	21.60b ± 1.23	107.01c ± 2.04	42.38a ± 2.21	63.33a ± 2.21
Broadcast	STR	24.22c ± 1.23	126.13d ± 2.04	56.74d ± 2.21	70.75c ± 2.21
P value		**0.000**	**0.000**	**0.000**	**0.000**

Values with different letters differ significantly.

Significant values are in bold.

## Discussion

### Chemical composition of soybean under phosphorus fertilization

Among protein feeds, soybean is characterized by the high biological value of its protein^10,16,44,45^. Soybean contains 5.6–11.5% water, 32–43.6% protein, 15.5–24.7% fat, and 4.5–6.4% DM ash^44,46^. In our study, the water content was less than 9%, CP 30–34%, EE 21–23%, and CA around 6–7% (Table 1). Agrotechnical factors had no significant effect on the level of protein in the seeds tested except for interaction between the studied factors (Table 1). According to Szwejkowska (2005)^47^, the protein content of legume seeds depends not only on the genetic characteristics of the variety, but also on the climatic conditions prevailing during the growing season and on agrotechnical factors, especially nitrogen fertilization. The CP values obtained in the experiment did not correspond with the results reported by Michalek and Borowski (2006)^48^, who reported that the amount in soybeans ranges from 37.9% to 44%. In our study, TP content was 29–33% and dependent on interaction between the examined factors. Bobrecka-Jamro et al. (2018)^49^ also suggest that the protein content of legumes depends not only on the genetic characteristics of the variety, but also on agrotechnical factors, particularly nitrogen. The differences, as in their study taking into account fertilization, did not affect protein content. According to Gaj et al. (2013)^50^, varying P doses had no significant effect on protein accumulation and gluten accumulation in the grain. In our study, P fertilization had no effect on protein content in the seeds.

The crude fat content of the analyzed seeds ranged from 21 to 23% (Table 1). These levels were comparable to the values provided by the producer and those adopted in standards^46,51,52^. The values obtained are comparable or even higher than the levels determined in other studies [Michalek et al. (2006)]^18^: 14.2–15.2% The average fat content of soybean seeds in our study is also confirmed in a study by Batista et al. (2015)^53^. Pasternakiewicz and Dzhugan (2009)^54^ reported that soybean seeds contain 18 to 21% crude fat (EE) on dry matter. It is widely known that the value of this parameter depends on temperature and humidity. Kumar et al. (2017)^55,56^ noted that higher oil content with increasing levels of P was due to the fact that phosphorus is one of the main components of fatty acids. In our study, EE content was in line with the content from the experiment conducted by Michalek (2000)^18^ and did not depend on P fertilization but on inteaction between the examined factors, and this content was much higher under STR fertilization band method and SUP broadcast method (Table 1).

One of the limiting factors to the use of feed raw materials in animal mixtures is CF. In the present study, the content of this component was 6–7% (Table 1). The values obtained are similar to the results obtained by Kozak et al. (2008)^57^, in their research study, CF content was determined at 5.5–5.8%. The CF result obtained was similar to the values obtained in the study by Jarecki and Bobrecka-Jamro (2015)^58^. According to Kozak et al. (2008)^57^, the content of this component in soybean seeds is determined by weather conditions.

There are some reports that the protein concentrations of seed are increased or decreased by P fertilization. Based on the available literature, the influence of P application on the protein concentration of soybean seed may be different depending on the P application level, soil moisture condition, and characteristics of the cultivar used in the study. Krueger et al. (2013)^59^ reported that protein concentrations in soybean seed were not affected by P fertilization. However, in general according to Abbasi et al. (2012)^11^ and Yin et al. (2016)^60^ protein concentrations are enhanced with increased P application rate^11,60^. Also, in a study conducted by Taliman et al. (2019)^61^ the protein concentration of seed was significantly higher under P fertilization.

### Amino acid composition of soybean under P fertilization

Commonly, 18 AA (essential and non-essential) are reported as components of soybean protein. ASP and GLU are the most abundant, accounting for about 12% and 18% of total AA, respectively; however, they are considered non-essential for humans and monogastric animals^55^. The content in our study was significantly higher under the influence of STR fertilization. Essential AA led to a relatively smaller portion of the seed, including LYS, THR, TYR, ILE, LEU, HIS, PHE, VAL, and the sulfur amino acids cysteine and MET^62^. In Kumar et al. (2017)^55^, an increase in phosphorus and sulfur significantly increased the content of cysteine. The increase in CYS content as a result of phosphorus can be attributed to the improvement in plant nutrition with sulfur and nitrogen. The combined application of phosphorus and sulfur increased the CYS content from 0.45% in the control to a maximum of 1.03%. In our study, P content did not change the CYS level. T

In contrast to our results, the interaction resulting from the combined application of P and sulfur increased the MET content from 0.40% in the control to a maximum of 0.98% in the control.

### Content of macro-and micronutrients of soybean under phosphorus fertilization

In a study by Szostak et al. (2020)^10^, analysis of the effect of year and fertilization on P content showed that the introduction of nitrogen at 30 kg ha^−1^ and 60 kg ha^−1^ (split application) significantly reduced the content of this P in soybeans. The effect of the 30 kg ha^−1^ dose depended on the application date. Phosphorus content significantly decreased after application of 30 kg ha^−1^ N at BBCH 73–75 in 2015 and before sowing in 2016. In our study, P fertilization caused changes in P content in the seeds. Struvite led to increased P content in the seed compared to SUP and control pots.

Phosphorus is relatively immobile in soil. Plants may have limited P uptake if P is too far from the roots or if root growth is stunted. In our study, the P content did not depend on fertilizer placement, and the higher values achieved with band fertilization were statistically insignificant; this confirmed the above thesis. Low soil moisture can reduce P availability and limit uptake^63^. In our research, P content was statistically the highest under STR fertilization.

The concentration of Fe and Mn in our study was affected by P fertilization. In our study, Zn content was at a similar level under SUP and STR fertilization, without significant differences (Table 8).

In Kumar et al. (2017)^55^, a higher manganese content (4.19 mg kg^−1^) in soybeans was recorded in soybeans when 45 kg sulfur and 90 kg P2O5 ha^–1^ was used compared to the other lower treatment combinations. In our research, we did not find any such relation with P fertilization. At higher P application levels, Kumar et al. (2017)^55^ noted a decrease in the Zn content in the seeds, which contrasted with our results.

## Conclusions

The working hypothesis partially showed that struvite fertilization modified the chemical composition as well as the biological value of protein and so the the null hypothesis should be completely rejected. Fertilization with STR appears to be comparable or even better than SUP due to the increase in EAA, as well as CA and P. In general, the interaction between factors caused an increase in most parameters concerning the nutritional value of soybean seeds; thus, STR could be a substitute for traditional P fertilizers. The use of STR resulted in an increase in some macronutrients and micronutrients essential for animal nutrition, such as P, Na, Ca, Fe, and Mn.

The results presented in this study indicate the need to conduct an assessment of the protein nutritional value of feed raw materials. The results of our own research confirm that chemical composition depends on the type (source) of fertilizer used and the method of its administration. The biological value of protein is determined not only by the content of CP but primarily by its amino acid composition, as demonstrated in our own research. Information is limited about the effects of phosphorus fertilization on soybean seed composition. Therefore, further research on this topic is necessary, especially field studies.

## Data Availability

All data generated or analyzed during this study are included in this published article.
